# Virtual reality interventions for cognitive remediation in severe mental illness

**DOI:** 10.1016/j.nsa.2025.105513

**Published:** 2025-02-21

**Authors:** Anna Julia Krupa

**Affiliations:** Department of Affective Disorders, Jagiellonian University Medical College, Kopernika St. 21a, 31-501, Krakow, Poland

**Keywords:** Virtual reality, Cognitive remediation, Schizophrenia, Depression, Bipolar disorder

Jespersen et al. reported on a proof-of-concept study testing the feasibility and effectiveness of virtual-reality (VR) assisted cognitive training in a mixed group of mood disorder and schizophrenia/schizotypal patients. In their work, patients participated in two VR immersed session of tasks reflecting every day life skills (planning and preparation of a meal) with a trained therapist and completed two home cooking assignments or continued treatment as usual over the course of one week. Their work indicated that VR intervention resulted in significant improvement in the global cognitive skills assessed with Cognition Assessment in Virtual Reality and in the processing speed domain (but not on other domains or traditional neurocognitive tests) (Jespersen et al., 2023).

Cognitive impairment is one of the core symptomatic dimensions of schizophrenia, that is often notable even before the psychosis onset, and it is one of the crucial determinants of every-day life functioning (Mucci et al., 2021). In fact, the levels of cognitive performance of people with schizophrenia are below the average of the general population and in the longitudinal perspective they remain relatively stable, unless addressed with a targeted treatment. The level of cognitive impairment related to schizophrenia varies between individuals, ranging from severe dysfunction to hardly any (Vita et al., 2024). As expected, cognitive remediation techniques have proven effective in improving neurocognition in schizophrenia, but they need to be implemented as part of more complex rehabilitation programs to help translate the improvement in cognition to better functional outcomes (Vita et al., 2024). It should be noted that cognitive impairment in schizophrenia encompasses not only neurocognition (attention, processing speed, working memory, learning memory, executive functions), socialcognition (perception and understanding of other's thoughts and emotions) but also metacognition, which is the ability to integrate psychological experiences into mental representations, to “think about thoughts” (Martiadis et al., 2023). This might explain, why the improvement of neurocognition is not enough to promote real-life patients functioning.

Jespersen et al. (2023) however, didn't include only psychosis spectrum subjects, but also patients with mood disorders. Indeed, persistent cognitive dysfunction is not reserved to psychotic disorders, although it seems to be most prominent in this clinical population. Chronic cognitive impairments are consistently noted in affective disorders (Jespersen et al., 2025), including metacognitive problems (Martiadis et al., 2023), and significantly limit patients functioning and quality of life (Jespersen et al., 2025). Much like in psychosis spectrum disorders, in mood disorder cognitive remediation improves neurocognitive performance which only partially translates to real-world functioning (Jespersen et al., 2025).

Patients with severe mental illness, whether it is a psychotic spectrum or a mood disorder, simply lack the ability to form more abstract concepts based on the performed cognitive tasks and later recognize their representations and opportunities to apply them in real life. And that is where the VR turns out to be useful, because, just as in the work by Jespersen and colleagues (Jespersen et al., 2023), VR offers a chance to train the cognitive skills in augmented environment simulating every-day life and fill the gaps in metacognition, by offering new metacognitive strategies for specific daily life tasks.

VR has more assets, like the chance to provide the patient with a safe and controllable environment, devoid of distracting stimulation, which could interfere with patients ability to engage with the intervention. On the other hand, the VR immersive environment can be stimulating just enough to keep the patient interested and avoid attention fatigue (Plencler et al., 2024). Obviously, because the VR environment can be adapted to individual patients needs, it provides a great opportunity to personalize the interventions and i.e. practice particular real-life tasks. Therefore VR holds great promises for the treatment of severe mental illness and the amount of reports on studies assessing the efficacy of VR in delivering interventions such as cognitive remediation, social skills training, mindfulness training etc. has rapidly increased in the past few years (Plencler et al., 2024; Vita et al., 2024).

Notably, as mentioned by Jespers et al. (Jespersen et al., 2023) patients are largely satisfied with the VR intervention and not only that, they felt *present*, *enjoyed* it and *felt entertained* by it. In this specific sample all participants completed the study and 75% performed the homework instructed. Although gathered during a very short intervention period, their observations are consistent with previous reports of patients feeling *intrigued* by VR (Kruk et al., 2023), or claiming that it promoted their motivation for training and they preferred it to conventional training (Rus-Calafell et al., 2018). The fact that VR makes the treatment more attractive to patients is vital in treatment of severe mental disorders, which is often plagued by low adherence and high rates of drop-outs (Jespersen et al., 2023).

Yet there are obstacles to be overcome such as trouble staying present due to discomfort caused by VR equipment (Rus-Calafell et al., 2018) and the need to adjust the VR immersive experience to individuals needs, as it was shown that in vulnerable subjects some VR scenarios might trigger paranoid beliefs (Kruk et al., 2023). Still, most patients tolerate the VR interventions well, reporting mild or none adverse effects (Kruk et al., 2023; Plencler et al., 2024; Rus-Calafell et al., 2018).

Furthermore, VR environments might be useful in providing cognitive examination, which would better approximate patients level of daily functioning than traditional neurocognitive tests. Jespersen et al. (2025) have recently reported that their VR cognitive assessment results were correlated with activities in daily life ability, while the neuropsychological test, interviewer or patients rated measures of functioning or subjective reports of cognitive performance were not (Jespersen et al., 2025).

For now VR interventions and tests spark enthusiasm, but need further verification. VR research is commonly characterized by methodological problems: small study samples, lack of randomization or blinding. This might be due to the nature of the VR intervention itself, as it requires a stable funding sufficient for collaboration of medical professionals and digital technology experts, equipment, and is not easy to blind (Selaskowski et al., 2024). Much needed double-blinded randomized clinical trials to examine the effects of VR based cognitive remediation in patients with mood and psychosis spectrum disorders are underway, among them the one by Jespersen and colleagues (Clinicalrials.gov Identifier: NCT06038955). Therefore, rigorous data exploring VR use in severe mental illness will likely be available soon.

This research did not receive any specific grant from funding agencies in the public, commercial, or not-for-profit sectors.

## Declaration of interests

The authors declare the following financial interests/personal relationships which may be considered as potential competing interests: Anna Julia Krupa reports a relationship with Angelini Pharma Poland that includes: consulting or advisory, funding grants, speaking and lecture fees, and travel reimbursement. Anna Julia Krupa reports a relationship with Lundbeck Poland that includes: travel reimbursement. Anna Julia Krupa reports a relationship with Swixx Biopharma Sp. z o.o. that includes: travel reimbursement. Anna Julia Krupa reports a relationship with Gedeon Richter Poland that includes: travel reimbursement. Anna Julia Krupa reports a relationship with Sandoz Polska Sp z oo that includes: travel reimbursement. If there are other authors, they declare that they have no known competing financial interests or personal relationships that could have appeared to influence the work reported in this paper.
